# Tiao He Yi Wei Granule, a Traditional Chinese Medicine, against Ethanol-Induced Gastric Ulcer in Mice

**DOI:** 10.1155/2015/647283

**Published:** 2015-12-08

**Authors:** Jinfu Yao

**Affiliations:** Changchun University of Chinese Medicine, Changchun 130117, China

## Abstract

Tiao He Yi Wei granule (DHYW), a traditional Chinese medicine, has been used for the treatment of gastric ulcer in clinical setting. The purpose of the present study was to investigate the possible effect of DHYW and explore the underlying mechanism against ethanol-induced gastric ulcer in mice. The model of ethanol-induced gastric ulcer in mice was induced by ethanol (0.2 mL/kg). Administration of DHYW at the doses of 250, 500 mg/kg body weight prior to the ethanol ingestion could effectively protect the stomach from ulceration. The gastric lesions were significantly ameliorated in the DHYW group compared with that in the model group. Treatment with DHYW markedly decreased the levels of interleukin-6 (IL-6), IL-1*β*, and tumor necrosis factor-*α* (TNF-*α*). In addition, DHYW treatment elevated myeloperoxidase (MPO) level in stomach, increased superoxide dismutase (SOD) activity, and decreased malonaldehyde (MDA) content in serum and stomach compared with those in the model group. DHYW significantly inhibited NF-*κ*B pathway expressions in the gastric mucosa ulcer group. Taken together, DHYW exerted a gastroprotective effect against gastric ulceration and the underlying mechanism might be associated with NF-*κ*B pathway.

## 1. Introduction

Peptic ulcer is one of the most pervasive gastrointestinal diseases which affect 4-5% people in the society [1]. The gastric ulcer is characterized by the reduction in blood flow, induction of oxidative stress, infiltration of neutrophils, and secretion of proinflammatory cytokines [2, 3]. A variety of factors such as* Helicobacter pylori* infection, alcohol consumption, smoking, excessive use of nonsteroidal anti-inflammatory drugs (NSAIDs), and psychological and physiological stress contribute to gastric ulcer [4]. Alcohol consumption increased the risk for major upper gastrointestinal bleeding [5]. As alcohol is one of the major abused agents, thus the alcohol-induced peptic ulcer is the common disorder of the gastrointestinal tract [6].

The nuclear factor kappa B (NF-*κ*B) signaling pathway is one of the most widely recognized intracellular signaling pathways in inflammatory responses [7]. Expressed in almost all cells, NF-*κ*B is involved in the mediation of various genes governing immune response and acute phase inflammatory reaction [8]. Various proinflammatory stimulus can lead to the activation of NF-*κ*B via the phosphorylation of inhibitors of *κ*B (I*κ*Bs) by the I*κ*B kinase (IKK) complex [9]. Then the free NF-*κ*B translocates into the nucleus and ultimately leads to the transcriptional activation of several proinflammatory mediators including TNF-*α*, IL-1*β*, and IL-6 [10].

Traditional Chinese medicine has been used for several centuries around the world and was considered as a main source of medicine [11, 12]. Tiao He Yi Wei granule (DHYW), a traditional Chinese medicine, contains eleven herbs, which are as follows:* Citrus medica *L., the fruit of* Polygonum orientale *L., the fruit of* Raphanus sativus *L.,* Fructus Amomi*, inner membrane of chicken gizzard,* Rhizoma Smilacis Glabrae*,* Atractylodes macrocephala*,* Lindera aggregata (Sims) Kosterm*,* Bletilla striata *(Thunb.)* Reichb. f., Citrus aurantium *L., and* Glycyrrhiza uralensis*. DHYW has been used for the treatment of gastric ulcer in clinical setting. However, few reports focused on the ethanol-induced gastric ulcer and the effects of DHYW. The aim of the study was to investigate the antigastric ulcer effects of DHYW on ethanol-induced mice and explore its possible mechanisms.

## 2. Materials and Methods

### 2.1. Reagents

*Citrus medica *L., the fruit of* Polygonum orientale *L., the fruit of* Raphanus sativus *L.,* Fructus Amomi*, inner membrane of chicken gizzard,* Rhizoma Smilacis Glabrae*,* Atractylodes macrocephala*,* Lindera aggregata (Sims) Kosterm*,* Bletilla striata *(Thunb.)* Reichb. f.*,* Citrus aurantium *L., and* Glycyrrhiza uralensis* were purchased from Jilin Drug Store (Changchun, China). As the positive control, omeprazole (OME) was supplied by Shanghai Xinyi Jiahua Pharmaceutical Company Limited (Shaanxi, China). All biochemical indicator kits were provided by the Institute of Jiancheng Bioengineering (Nanjing, China). The enzyme-linked immunosorbent assay (ELISA) kits for determination of IL-6, IL-1*β*, and TNF-*α* were produced by BioLegend (San Diego, CA, USA). All antibodies were supplied by Cell Signaling Technology.

### 2.2. Animals

A total of 50 female specific pathogen-free female BALB/c mice, aged 6–8 weeks, were obtained from the Center of Experimental Animals of Changchun University of Chinese Medicine (Changchun, China). Mice were kept in an animal facility under standard laboratory conditions for 1 week prior to the experiments and provided with water and standard chow* ad libitum*. All experimental procedures were performed in accordance with the NIH Guidelines for the Care and Use of Laboratory Animals and the National Animal Welfare Law of China.

### 2.3. HPLC Analysis of DHYW

An agilent 1100 liquid chromatography system (Agilent, Santa Clara, California, USA), equipped with a quaternary solvent delivery system, an autosampler and DAD detector, was used. A Zorbax Extend-C18 (250 mm × 4.6 mm, 5 *μ*m) column connected with a Zorbax Extend guard column (20 mm × 4.6 mm, 5 *μ*m) was used. The column temperature was set at 25°C. The mobile phase consisted of (A) acetonitrile and (B) 0.1% formic acid-water (*V*/*V*) using a linear gradient elution of 5%−55% A at 0−5 min, 55%−80% A at 6−10 min, 80%−95% A at 11−15 min, and 95%−5% A at 16−17 min. The flow rate was 1.0 mL·min^−1^ and 10 *μ*L of samples was injected. Ultraviolet detection was performed on a UV-2450 spectrometer (Shimadzu, Kyoto, Japan). The chromatograms were monitored at 280 nm and the absorption spectra of compounds were recorded from 200 to 400 nm.

### 2.4. Induction of Gastric Ulcer and Treatment

Five groups of mice were assigned: groups 1 and 2 were orally given PBS (vehicle). Group 3 was administered omeprazole (OME) 20 mg/kg; Group 4 and 5 were given DHYW (250 mg/kg and 500 mg/kg, resp.). After an additional hour, the mice in groups 2–5 intragastrically received ethanol (0.2 mL/kg) while group 1 received PBS at the same volume. Mice were sacrificed after 4 h. The serum and stomach tissues were harvested for the further studies.

### 2.5. Determinations of MPO in Stomach Tissue and SOD and MDA in Serum and Stomach

At the end of the experiment, the blood was centrifuged at 3000 rpm for 8 min and then the serum samples were stored at −80°C for pending tests. Stomach tissues were homogenized with cold normal saline and centrifuged at 12,000 rpm for 10 min at 4°C; the supernatant of the homogenate was collected and stored at −80°C. The protein content of stomach sample was determined using a BCA protein assay kit. The levels of SOD and MDA in serum and stomach as well as MPO activity in stomach tissue were determined using test kits purchased from Nanjing Jiancheng Bioengineering Institute (China, Nanjing) according to the manufacturer's protocols.

### 2.6. Determinations of the Levels of IL-1*β*, IL-6, and TNF-*α* in Serum

IL-1*β*, IL-6, and TNF-*α* levels in serum were determined with ELISA kits purchased from BioLegend (San Diego, CA, USA). All procedures were carried out according to the manual. Then the absorbance of each well was read at 450 nm by a microplate spectrophotometer.

### 2.7. Histological Analysis

Stomach sample removed from each mouse was fixed in 10% buffered formalin for more than 48 h. After dehydrating in graded alcohol and embedding in paraffin wax, the sections were cut to a thickness of 4 *μ*m. Then the samples were stained with hematoxylin and eosin (H&E) for histological evaluation. The pathological changes in the gastric tissues were observed under a light microscope.

### 2.8. Western Blot of NF-*κ*B Pathway

The stomach tissues were homogenized, washed with PBS, and lysed in a RIPA buffer (Beyotime, Nanjing, China). After the determination of the protein concentration using an enhanced BCA kit (Beyotime, China), the samples were loaded to 10% SDS-PAGE gels and then electrotransferred to a polyvinylidene difluoride membrane (Millipore, MA, USA). The blots were incubated with the appropriate concentrations of specific antibodies at 4°C overnight. After washing, the blots were incubated with horseradish peroxidase-conjugated second antibodies. The membrane was stripped and reblotted to verify the equal loading of protein in each lane. Quantification of protein expression was normalized to GAPDH or Histone H3 using a densitometer (Imaging System).

### 2.9. Statistical Analysis

All values were expressed as the means ± SDs and analyzed with one-way analysis of variance (ANOVA) with Tukey's multiple comparison test. *P* < 0.05 was considered significant and *P* < 0.01 was considered to be statistically very significant.

## 3. Results

### 3.1. HPLC Analysis of DHYW

**Figure 1:**
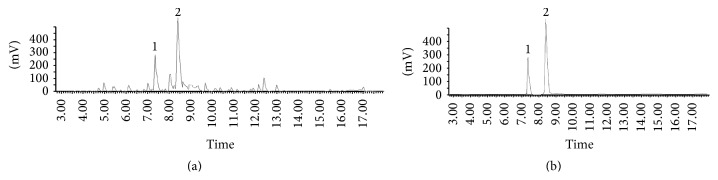
HPLC analysis of DHYW. (a) Sample of DHYW. (b) Sample of hesperidin and glycyrrhizic acid. 1: hesperidin, 2: glycyrrhizic acid.

As shown in Figure 1, two compounds which have gastroprotective action were determinate: one is hesperidin; another is glycyrrhizic acid, and the contents are 0.43 mg/g and 0.69 mg/g, respectively.

### 3.2. Effect of DHYW on Gastric Lesions

**Figure 2:**
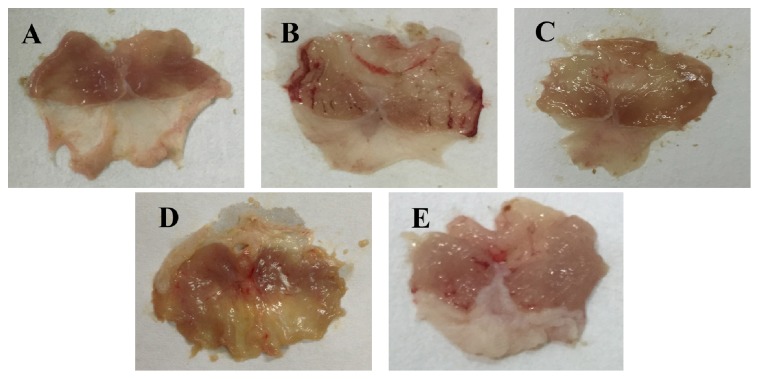
Macroscopic evaluation of the effect of DHYW on ethanol-induced gastric ulcer. Mice were intragastrically administered with DHYW (250 mg/kg, 500 mg/kg) or OME (20 mg/kg). 1 h later, the mice were intragastrically given ethanol (0.2 mL/kg) and were sacrificed at 4 h after ethanol challenge. These are representative photos from (A) control group, (B) ethanol group, (C) ethanol + OME (20 mg/kg) group, (D) ethanol + DHYW (250 mg/kg) group, and (E) ethanol + DHYW (500 mg/kg) group.

Acute gastric lesions were induced by intragastric administration of ethanol. As revealed in Figure 2, the ethanol-stimulated mice presented extensive elongated thick, dark red and black bands of hemorrhagic lesions on the glandular part of the stomach compared with that of control animals. By contrast, treatment with DHYW (250 and 500 mg/kg) or OME effectively attenuated the severe gastric mucosal damage caused by ethanol.

### 3.3. Effect of DHYW on SOD and MDA Levels in the Ethanol-Treated Mice

**Figure 3:**
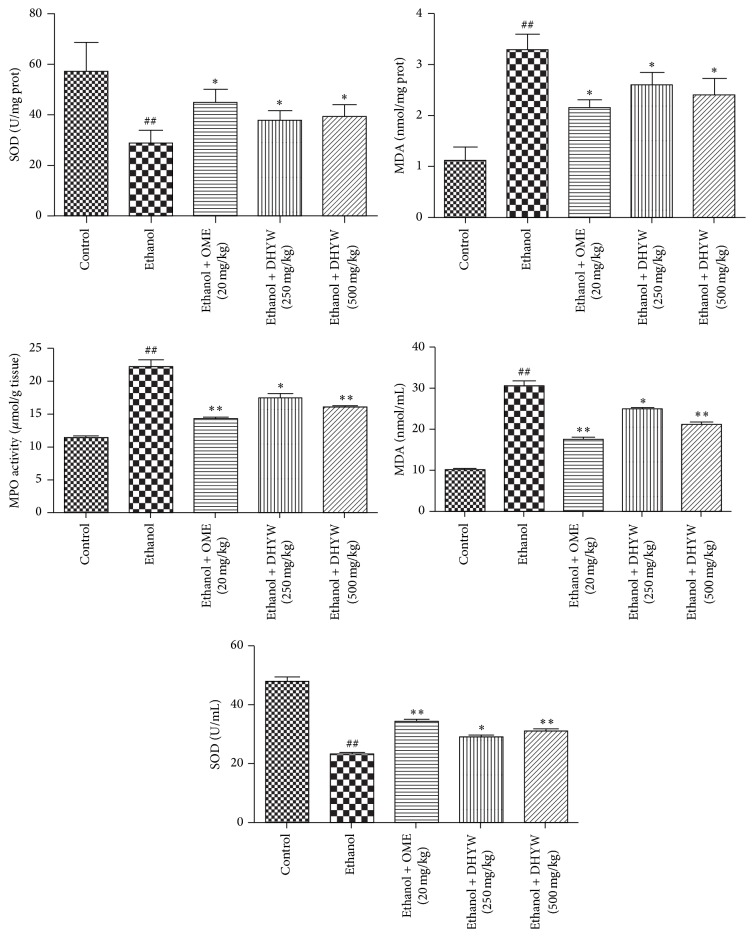
Effects of EVD on SOD and MDA levels in the serum and MPO activity in stomach tissue of the ethanol-treated mice. Mice were intragastrically administered with DHYW (250 mg/kg, 500 mg/kg) or OME (20 mg/kg). 1 h later, the mice were intragastrically given ethanol (0.2 mL/kg) and were sacrificed at 4 h after ethanol challenge. Values are expressed as means ± SEM. Compared with control: ^#^ *P* < 0.05, ^##^ *P* < 0.01; compared with model: ^*∗*^ *P* < 0.05, ^*∗∗*^ *P* < 0.01.

To evaluate the effect of DHYW on lipid peroxidation in gastric ulcer, the levels of SOD and MDA were measured using commercial kits. As revealed in Figure 3, ethanol stimulation significantly declined the SOD activity in serum and stomach tissues, while DHYW (250 and 500 mg/kg) treatment effectively restored the level of SOD. In addition, exposure to ethanol displayed strikingly high MDA contents in both serum and stomach tissue, whereas the oral administration with DHYW (250 and 500 mg/kg) significantly ameliorated these situations. The experimental data clearly demonstrated that DHYW was capable of mediating the oxidative stress in ethanol-stimulated gastric ulcer.

### 3.4. Effects of DHYW on Inflammatory Cytokines

**Figure 4:**
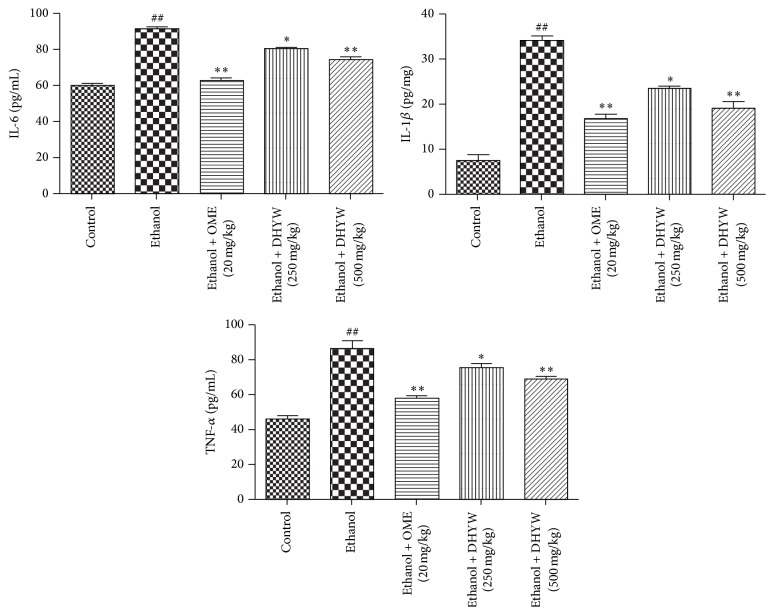
Effects of DHYW on inflammatory cytokine. Mice were intragastrically administered with DHYW (250 mg/kg, 500 mg/kg) or OME (20 mg/kg). 1 h later, the mice were intragastrically given ethanol (0.2 mL/kg) and were sacrificed at 4 h after ethanol challenge. Values are expressed as means ± SEM. Compared with control: ^#^ *P* < 0.05, ^##^ *P* < 0.01; compared with model: ^*∗*^ *P* < 0.05, ^*∗∗*^ *P* < 0.01.

Next, we detected the levels of TNF-*α*, IL-6, and IL-1*β* in serum by ELISA kits to confirm the anti-inflammatory properties. As expected, inflammatory cytokines were remarkably increased in serum of the ethanol-induced mice compared with those in control mice. However, treatment with DHYW (500 mg/kg) and OME (20 mg/kg) significantly reduced the levels of the TNF-*α*, IL-6, and IL-1*β*, which were slightly more potent than those in DHYW (250 mg/kg) group. The results displayed that DHYW inhibited the generations of inflammatory cytokines in gastric ulcer animals (Figure 4).

### 3.5. Histological Evaluations

**Figure 5:**
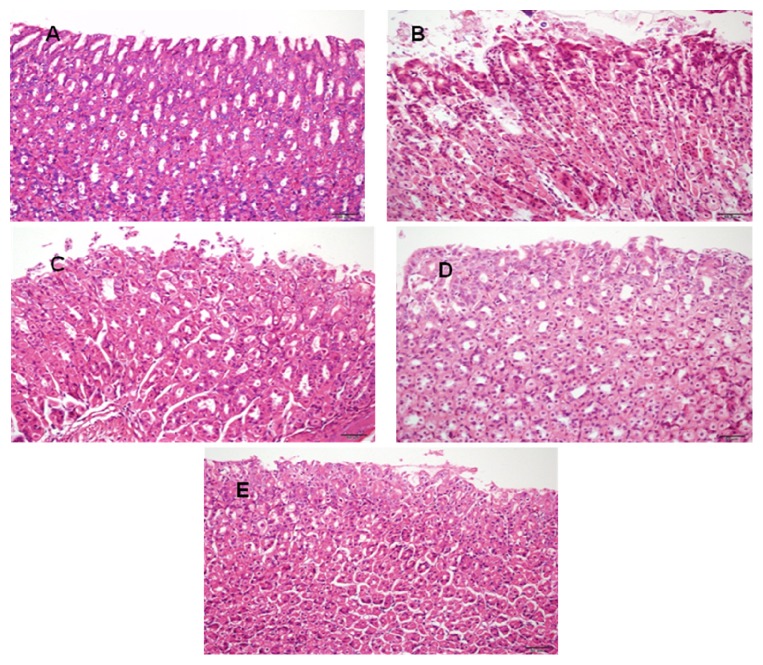
Effects of DHYW on ethanol-stimulated histopathologic changes in stomach tissues. (A) The stomach section from the control mice; (B) the stomach section from the mice administered with ethanol; (C) the stomach section from the mice administered with OME (20 mg/kg) and ethanol; (D) the stomach section from the mice administered with DHYW (250 mg/kg) and ethanol; and (E) the stomach section from the mice administered with DHYW (500 mg/kg) and ethanol.

The histopathology of stomach tissues also confirmed the protective effect of DHYW. As illustrated in Figure 5, animals with ethanol-induced ulcer showed more extensive damage to the gastric mucosa, edema, and leucocyte infiltration compared with those of control mice. On the contrary, treatments with DHYW (250 and 500 mg/kg) and OME proved comparatively better protection of the gastric mucosa as evidenced by less submucosal edema, reduction in ulcer area, and suppression of leucocyte infiltration. The analytical data displayed that DHYW obviously attenuated the histopathology situation in ethanol-induced gastric ulcer.

### 3.6. Effects of DHYW on NF-*κ*B

**Figure 6:**
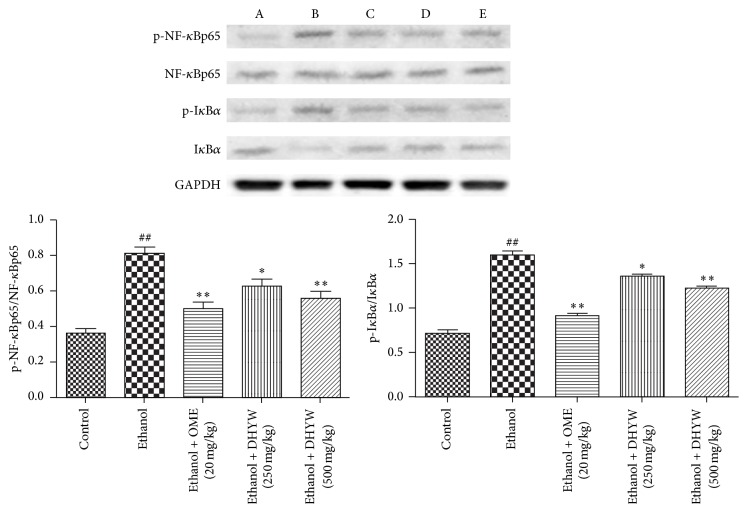
Effects of DHYW on NF-*κ*B pathway. Mice were intragastrically administered with DHYW (200 mg/kg, 500 mg/kg) or OME (20 mg/kg). 1 h later, the mice were intragastrically given ethanol (0.2 mL/kg) and were sacrificed at 4 h after ethanol challenge. (A) control group, (B) ethanol group, (C) ethanol + OME (20 mg/kg) group, (D) ethanol + DHYW (250 mg/kg) group, and (E) ethanol + DHYW (500 mg/kg) group. Values are expressed as means ± SEM. Compared with control: ^#^ *P* < 0.05, ^##^ *P* < 0.01; compared with model: ^*∗*^ *P* < 0.05, ^*∗∗*^ *P* < 0.01.

To further detect the underlying mechanism of DHYW, we evaluated the protein expression of NF-*κ*B signaling. As depicted in Figure 6, ethanol significantly upregulated the expressions of p-I*κ*B and p-NF-*κ*B. However, the administrations of DHYW (500 mg/kg) and OME effectively inhibited the phosphorylations of I*κ*B and NF-*κ*B, which were slightly more potent than that of DHYW (250 mg/kg) group. Our results suggested that DHYW might exert its gastroprotective effects via the activation of NF-*κ*B signaling.

## 4. Discussion

With the risk of human health in daily life, gastrointestinal problems have been a global issue which was always ignored at early stage. In general, gastric ulcer is a common disease triggered by various etiologies including free oxygen radicals. Ethanol is widely acknowledged as an abused agent to gastric ulceration [13]. Thus, the gastric ulcer model induced by ethanol in mice was applied to examine the effect of the compounds against gastric ulcer in our study. As the incentive of gastric ulcer, ethanol ingestion in the model mice led to an obvious pathological process compared with that of control mice, which was elucidated by acute gastric mucosal lesions through the neutrophil infiltration, release of proinflammatory cytokines, and the expression of nuclear factor-*κ*B (NF-*κ*B). Despite taking into consideration the relation between efficacy and dose, omeprazole (OME) was proved to possess a more marked inhibition against gastric ulcer in comparison with DHYW. However, the potential role of DHYW was also indicated in the improvement of gastrointestinal problems. On the basis of successful model method, the purpose of the present study was to evaluate the antiulcer activity of DHYW using an ethanol-challenged experimental gastric ulcer model and investigate its potential mechanisms. The above results indicated that DHYW could reverse the lesion of gastric mucosa from ethanol induced ulceration to a degree via anti-inflammatory and antioxidative effects which are probably mediated by inhibiting NF-*κ*B pathway activation.

There is growing evidence that ethanol-induced gastric mucosal injury is highly related to the increased ROS level. ROS mainly comes from neutrophils which were driven by MPO under inflammatory circumstance [14, 15]. Neutrophil infiltration takes the responsibility for prompting reactions in the process of injury by releasing and aggregating tissue-disrupting events in tissues [16, 17]. Therefore, MPO could be treated as a marker to estimate the accumulation of neutrophil infiltration into the gastric mucosal tissues. On the other hand, organisms' enzymatic and nonenzymatic defenses including SOD protect the host against the ROS-induced lipid peroxidation [18]. Generally, SOD exerts multifaceted physiological activities including anti-inflammatory and antioxidant effects [19]. Meanwhile, MDA is the end-product of polyunsaturated fatty acid, which is often used as an indicator for evaluating the lipid peroxidation in gastric mucosal [20, 21]. Our data supported a critical role of oxidative stress in the pathogenesis of the ethanol-induced gastric ulcer. Nevertheless, pretreatment with DHYW resulted in significant increases in the activity of SOD, as well as the decreases in MDA and MPO levels. Hence, our study revealed that DHYW potentially exerted gastroprotective properties alleviating neutrophil infiltration and lipid peroxidation induced by growing ROS level through an antioxidative mechanism, as likewise evidenced by ameliorative consequences in the histological evaluations.

Despite the widely accepted notion that alcohol abuse leads to detrimental consequences in the gastrointestinal tract, the underlying mechanisms still remain obscure. Evidence has emerged indicating that ethanol ingestion may activate the innate immune system to change the levels of proinflammatory cytokines including TNF-*α*, IL-6, and IL-1*β* [22]. The generation of inflammatory mediators also plays an important role for the mechanisms of lesions in providing an inflammatory circumstance to facilitate the development of acute gastric mucosal lesions [23]. Previous findings implicated that the productions of proinflammatory cytokines remarkably increased in the serum and gastric tissue of the ethanol-induced ulcer [24]. As a representative inflammatory cytokine with pleiotropic functions, TNF-*α* is closely associated with the progression of inflammatory disorder and the activation of relevant proinflammatory cytokines such as IL-6 and IL-1*β* [25]. Previous research showed an aberrant high level of IL-6 in the serum of mice with inflammatory diseases including severe mucosal inflammation [26]. Therefore, the possible alterations of TNF-*α*, IL-6, and IL-1*β* contents were investigated. Our experimental results indicated that the serum IL-6 and TNF-*α* levels were significantly increased due to the necrotizing effects of ethanol. However, the inflammatory status had been favorably reduced in the animals pretreated with DHYW, which suggested that the effect of DHYW treatment the effectively inhibited in the generation of proinflammatory cytokines.

NF-*κ*B, well known to mediate the expression of proinflammatory molecules, is a vital transcription factor that regulates many immune and inflammatory processes [27]. NF-*κ*B has been extensively investigated for cytokine regulation and oxidative stress mediation [28]. It was noteworthy that NF-*κ*B pathway was involved in the pathogenesis of ethanol-induced gastric lesion [29]. Therefore, the inhibition of phosphorylated I*κ*B and NF-*κ*B in the gastric tissue might be an important element in the protective effect of DHYW on ethanol-induced gastric lesions. This assumption was confirmed by the suppression of NF-*κ*B signaling observed in animals treated with DHYW in response to ethanol.

In conclusion, our data suggested that DHYW might effectively ameliorate ethanol-induced gastric ulcer by attenuating the inflammatory and oxidative conditions via the NF-*κ*B signaling pathway. Thus, these results supported the notion that DHYW was beneficial for the treatment of ethanol-induced gastric ulcer. Its exact mechanism and clinical application need further investigation in the future.
